# *Bacillus anthracis* Protective Antigen Kinetics in Inhalation Spore-Challenged Untreated or Levofloxacin/Raxibacumab-Treated New Zealand White Rabbits

**DOI:** 10.3390/toxins5010120

**Published:** 2013-01-14

**Authors:** Alfred Corey, Thi-Sau Migone, Sally Bolmer, Michele Fiscella, Chris Ward, Cecil Chen, Gabriel Meister

**Affiliations:** 1 Human Genome Sciences, Inc., 14200 Shady Grove Road, Rockville, MD 20850, USA; E-Mails: thi_migone@hgsi.com (T.-S.M.); sally_bolmer@hgsi.com (S.B.); mfissh@gmail.com (M.F.); c-ward@hotmail.com (C.W.); cckchen@verizon.net (C.C.); 2 Battelle Biomedical Research Center, 505 King Ave., JM-7, Columbus, OH 43201, USA; E-Mail: meisterg@battelle.org

**Keywords:** protective antigen, kinetics, anthrax, raxibacumab

## Abstract

Inhaled *Bacillus anthracis* spores germinate and the subsequent vegetative growth results in bacteremia and toxin production. Anthrax toxin is tripartite: the lethal factor and edema factor are enzymatic moieties, while the protective antigen (PA) binds to cell receptors and the enzymatic moieties. Antibiotics can control *B. anthracis* bacteremia, whereas raxibacumab binds PA and blocks lethal toxin effects. This study assessed plasma PA kinetics in rabbits following an inhaled *B. anthracis* spore challenge. Additionally, at 84 h post-challenge, 42% of challenged rabbits that had survived were treated with either levofloxacin/placebo or levofloxacin/raxibacumab. The profiles were modeled using a modified Gompertz/second exponential growth phase model in untreated rabbits, with added monoexponential PA elimination in treated rabbits. Shorter survival times were related to a higher plateau and a faster increase in PA levels. PA elimination half-lives were 10 and 19 h for the levofloxacin/placebo and levofloxacin/raxibacumab groups, respectively, with the difference attributable to persistent circulating PA-raxibacumab complex. PA kinetics were similar between untreated and treated rabbits, with one exception: treated rabbits had a plateau phase nearly twice as long as that for untreated rabbits. Treated rabbits that succumbed to disease had higher plateau PA levels and shorter plateau duration than surviving treated rabbits.

## 1. Introduction

*Bacillus anthracis* causes anthrax [1], and its endospores have been developed as biological weapons [2]. Human inhalational anthrax mortality ranges from 45% to 80% [1,2,3]. Although antibiotics can be used to treat *B. anthracis* infection [2], therapeutics directed against the anthrax toxin are needed. The anthrax toxin is a tripartite toxin: two factors, the lethal factor (LF) and edema factor (EF), have enzymatic activities, while the protective antigen (PA) binds to cell receptors and then binds and translocates LF and EF into the cell. Inhibiting binding of PA to its cell receptors blocks the binding of LF and EF, and thus also the internalization of the anthrax toxin [4,5].

Clinical presentation of inhalational anthrax is similar in non-human primates, rabbits, and humans [6,7,8,9]. Animal studies provided the basis for approval of antibiotics and anthrax vaccine adsorbed for use in humans [10,11]. Raxibacumab is a fully human IgG_1_ monoclonal antibody against PA [12], and is efficacious in animal models of inhalational anthrax [13]. Justification of a human raxibacumab dose under the “Animal Rule” (21 CFR 601.90-96) [13,14] requires consideration of efficacious doses in animal models and attaining similar levels in humans as in animals. In addition, it is necessary to consider PA kinetics in the animal models to assure that the selected human dose would provide raxibacumab levels at least equimolar to the highest expected PA exposure.

Prior studies have shown that raxibacumab is efficacious in the treatment of inhalation anthrax in the post-exposure prophylaxis setting, as well as when administered at the onset of disease [13]. In those studies, onset of disease was determined by either the first significant increase in body temperature or the first detection of measurable serum or plasma PA levels, since those clinical parameters tend to be coincident with detectable bacteremia [13]. This study sought to determine the added benefit of raxibacumab combined with antibiotic treatment compared with antibiotic treatment alone and to define PA kinetics in the rabbit model of inhalational anthrax, when treatment was initiated late in the course of disease.

## 2. Materials and Methods

The in-life portions of this good laboratory practice study were conducted at the Battelle Biomedical Research Center (West Jefferson, OH, USA). The study was approved by Battelle’s institutional animal care and use committee. The assays for plasma PA concentrations and PA kinetic analyses were performed at Human Genome Sciences, Inc. (HGS; Rockville, MD, USA).

### 2.1. Animals

Pathogen-free New Zealand white rabbits surgically implanted with vascular access ports (VAP) were supplied by Covance Research Products, Inc. (Denver, PA, USA). Of the 210 rabbits (105 males and 105 females) supplied, 180 were required for the study, with the extra animals available as replacements before the spore challenge, if required. No rabbits were replaced after the spore challenge. After a quarantine period (minimum of 7 days), rabbits that were in good health, free of malformations, and exhibited no signs of clinical disease were randomly selected for the study. Rabbit age was not a criterion for placement in this study.

### 2.2. Test Articles

#### 2.2.1. Levofloxacin

Levaquin^®^ Oral Solution (levofloxacin 25 mg/mL, Lot No. AEB2V00) was administered intragastically (IG) as supplied, without dilution.

#### 2.2.2. Raxibacumab

Raxibacumab (Lot 71128, 50 mg/mL) and raxibacumab vehicle (Lot 71043, formulation buffer for placebo) were produced at HGS as ready-to-use sterile liquid formulations and were stored at 2 to 8 °C prior to use. Raxibacumab vehicle contains 0.13 mg/mL citric acid, 2.8 mg/mL sodium citrate, 10 mg/mL sucrose, 18 mg/mL glycine, and 0.2 mg/mL polysorbate 80 at pH 6.5.

Prior to administration, raxibacumab or vehicle control were aseptically transferred to individual dosing vials according to a randomization scheme, and the vials were stored at 2 to 8 °C prior to dosing. The study director and technicians were blinded to raxibacumab or placebo administered to each rabbit.

### 2.3. Study Design

The study was conducted in 3 sets of challenge days, with 70 rabbits representing a challenge set. Rabbits from each challenge set were randomized to 1 of 2 challenge days such that 30 rabbits (15 males and 15 females) were spore challenged on each day, and the remaining 10 rabbits were a replacement group. The challenge order within each challenge day was also randomized.

On Study Day 0, rabbits were individually challenged with a targeted aerosol 200 times the median lethal dose (LD_50_, 105,000 colony forming units [9]) of *B. anthracis* (Ames strain) spores in a plethysmography chamber and a Class III cabinet system. The aerosol challenge duration was based upon an estimated aerosol challenge concentration and a cumulative minute volume gathered “real” time throughout the exposure.

Treatments were initiated at a fixed time of 84 h (±4 h) *post* the median challenge time for a group of animals spore challenged on the same day. Levofloxacin was administered by gastric intubation, followed by intravenous (IV) injection of raxibacumab or placebo (raxibacumab buffer), such that there were 2 treatment groups: levofloxacin alone or levofloxacin in combination with raxibacumab.

The 76 rabbits that survived to the designated treatment time were randomly assigned to the 2 treatment groups. All treated rabbits were administered 50 mg/kg of levofloxacin IG once daily (qd) for 3 consecutive doses (2 mL/kg). Immediately after administration of the first levofloxacin dose, rabbits also received either a single IV 40 mg/kg raxibacumab or placebo dose (0.8 mL/kg) via the VAP or a marginal ear vein. Table 1 provides a summary of the characteristics and disposition of the rabbits in this study.

**Table 1:** Summary of rabbit characteristics and disposition in this study.

	Sex	Weight	Spore Challenge
		(kg) ^1^	(LD_50_) ^1^
Untreated (*n* = 104)	54 Males	3.16	189
	50 Females	±0.25	±44
Treated—Levofloxacin (*n* = 37)	18 Males	3.12	174
	19 Females	±0.24	±43
Treated—Levofloxacin + Raxibacumab (*n* = 39)	18 Males	3.1	197
	21 Females	±0.24	±49

^1^ Mean and standard deviation are presented.

### 2.4. Specimen Collection

Blood samples for plasma PA analysis were taken from the VAP, the medial auricular artery, or the marginal ear vein into tubes containing ethylenediaminetetraacetic acid as an anticoagulant. Blood specimens were collected from all rabbits prior to spore challenge, as well as from all surviving rabbits at 12, 24, 36, 48, 60, and 72 h after spore challenge. For all treated rabbits, additional blood specimens were collected just prior to the first levofloxacin dose; at 5 min and 8 h after the raxibacumab dose; at 2 h after each levofloxacin dose; at 24 h after the first and second levofloxacin doses; at 2 and 3 days after the third levofloxacin dose; and at 7, 14, 21, and 28 days after the raxibacumab dose. When feasible, a terminal blood sample was taken just prior to euthanasia for animals that were judged to be moribund. Blood specimens collected at 7, 14, 21, and 28 days *post* raxibacumab dose, as well as terminal specimens, were not collected from the VAP. Blood specimens were centrifuged, and the plasma was harvested. The plasma was filtered, tested for sterility, and stored at ≤−70 °C prior to shipment to HGS for assay. Bacteremia was assessed by culture at all collection times, with the exceptions of 5 min and 8 h *post* raxibacumab dose. Quantitative bacteremia was determined at 24 h post-challenge, immediately prior to the first treatment (84 h), 24 h after the first levofloxacin dose (prior to the second levofloxacin dose), and 2 days *post* the third levofloxacin dose.

### 2.5. Bioanalytical Method

Samples were stored at ≤−70 °C at HGS. Just prior to assay, samples were thawed and working aliquots taken. Plasma total (free and raxibacumab-bound) PA concentrations were determined using an electrochemiluminescence (ECL)-based bridging assay. In brief, diluted plasma samples in duplicate wells were combined with rabbit polyclonal antibody (pAb) anti-PA-biotin (capture) and rabbit anti-PA pAb labeled with Meso Scale Discovery SULFOTAG™, an ECL label (detector), and allowed to equilibrate in a streptavidin-coated assay plate. Following equilibration, plates were washed and read for ECL counts. The concentration of PA in plasma samples was interpolated from a reference standard curve. The assay run had to meet system suitability criteria for the results to be considered valid. Samples were diluted 1:4 or 1:500, depending on the expected PA concentration. If samples were out of range at either dilution, they were then repeated at the alternative dilution. For each dilution, 3 quality controls (QC) were run twice on each plate (6 total per plate). For the 1:4 dilution, 820, 24, and 1.5 ng/mL were used. For the 1:500 dilution, 102500, 3000, 187.5 ng/mL were used. Four out of the six QC samples had to recover between 75% and 125% with a CV ≤ 20%. At least one QC of each QC level must have met this criterion. Individual samples had to have a CV ≤ 20% to be accepted. The lower and upper limits of quantitation were 0.5 ng/mL and 136,500 ng/mL of PA in 100% rabbit plasma, respectively. Accuracy and precision was measured at 0.5, 1.5, 24, and 820 ng/mL. Accuracy values of 96%, 115%, 108%, and 102%, respectively, were observed for each spike. Precision values of 13%, 11%, 6%, and 8%, respectively, were observed for each spike. Both the reference standard curve and QC were prepared with recombinant PA (83 kDa) with an estimated purity of 97% (based on reverse phase high performance liquid chromatography). PA was prepared based on the method of Laird *et al.* [15]. The antibodies in this assay were only evaluated against this recombinant PA.

### 2.6. Data Analysis

Actual times *post* spore challenge, actual magnitude of spore challenge, and actual dose times were used for the analyses. PA concentrations below the lower limit of quantitation were treated as zero. The PA concentration-time data was divided into 2 subgroups for analysis. The first subgroup comprised those rabbits that were subjected to the spore challenge but did not survive until the 84 h post-challenge treatment time (*i.e.*, untreated rabbits). The other subgroup comprised those rabbits that were subjected to the spore challenge, survived until the 84 h post-challenge treatment time, and were administered levofloxacin and either raxibacumab or placebo (*i.e.*, treated rabbits). The plasma PA concentration-time data for both subgroups were analyzed using the NONMEM software (Version VI 1.3).

#### 2.6.1. PA Kinetics in Untreated Animals

Systemic PA levels have been shown to correlate with *B. anthracis* septicemia, reflecting both the onset and magnitude [16,17]. In a rabbit study of the natural history of inhalational anthrax [17], plasma PA concentration-time profiles comprised several phases: an initial lag phase, followed by a rapid rise, which is followed in turn by a plateau period, then a second phase of rapidly increasing levels, with evidence of a terminal plateau phase. The first 3 phases of the profile are consistent with the Gompertz equation (Equation 1) that is used to describe biological growth [18], which displays a lag phase, followed by an exponential growth phase, which then approaches an asymptote:

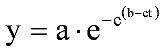
(1)
where *y* is the number of bacteria present at time *t*, and *a*, *b*, and *c* are constants.

Zwietering *et al.* [19] published a modification of the Gompertz equation (Equation 2), in which the model was expressed in terms of parameters with biological meaning:

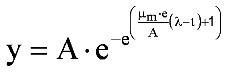
(2)
where y = ln(N/N_0_) and A = ln(N_∞_/N_0_). *N* is the number of bacteria present at time *t*, *N_0_* is the number of bacteria initially present at time 0, *N_∞_* is the number of bacteria present at infinite time (the asymptotic phase), μ_m_ is the maximum specific growth rate, and λ is the lag time.

Surrogate measures for bacterial numbers, such as absorbance of light at 620 nm by liquid bacterial growth medium, have been used in the Gompertz model [20,21]. Since systemic PA concentrations correlate with the time course and magnitude of *B. anthracis* septicemia [16,17], plasma PA levels could be applied in the Gompertz model. Many bacteria display a diauxic growth pattern that consists of an initial phase as described by the Gompertz model; following establishment of the asymptotic plateau, a new exponential growth phase begins, followed by a terminal asymptotic phase. In essence, a diauxic growth curve looks like 1 Gompertz growth curve followed by another. Diauxic growth curves have been noted in circumstances where bacterial growth is dependent on preferential utilization of 1 of 2 or more available nutrients [22]. Liquori *et al.* [23] modeled diauxic growth as the sum of 2 separate weighted growth functions, as in Equation 3:

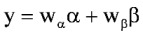
(3)
where α and β are the functions describing the 2 phases of the diauxic growth curve, and *w_α_* and *w_β_* are weights applied to the 2 functions; the sum of the 2 weights is constrained to equal 1.

That approach was applied to describe diauxic plasma PA concentration-time profiles [17], applying the modified Gompertz equation [19] in the following system of equations (Equation 3, plus Equation 4 to Equation 6):

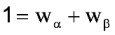
(4)

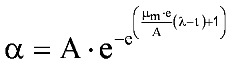
(5)

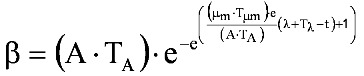
(6)
where *T_A_*, *T_μm_*, and *T_λ_* are the factors by which A, μ_m_, and λ in the β phase differ from those in the α phase.

In the present study, untreated rabbits exhibited at least 1 or more of the following phases of the diauxic Gompertz model: initial lag phase, first rising phase, plateau phase, and second rising phase, but no rabbits had evidence of the terminal plateau phase in their profile. Since there was no evidence of a terminal plateau phase, a composite modified Gompertz/second exponential growth model was used to describe plasma PA kinetics. That model consisted of the modified Gompertz model combined with a second exponential rising phase that occurs after a second lag phase, and is described by the following set of equations:

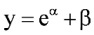
(7)

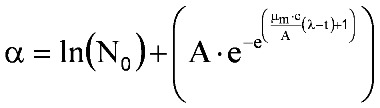
(8)

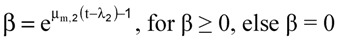
(9)
where μ_m,2_ is the maximum specific growth rate for the second rising phase and λ_2_ is the lag time for the second rising phase.

Body weight, sex, age, size of spore challenge, duration of spore challenge, and time to first bacteremia by culture (TBAC) were evaluated as potential covariates.

#### 2.6.2. PA Kinetics in Treated Animals

In the present study, it was observed that plasma PA concentration-time profiles in treated animals generally showed an initial rise, but before or shortly after the second rising phase was attained, plasma PA concentrations began to decline, with the decline of plasma PA levels generally coinciding with the sterilization of bacteremia. Hence, it was expected that, for treated rabbits, PA levels would initially follow the same composite modified Gompertz/second exponential growth model described by Equation 7 through 9, but with the addition of a monoexponential elimination phase that starts after treatment. This modified model can be expressed as follows:

For times *t* ≤ *t_tx_*, Equation 7 through 9 apply; For times *t* > *t_tx_*;

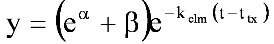
(10)
where α and β are evaluated at the treatment time (*t_tx_*), and k_elm_ is the first order elimination rate constant. Although this model assumes that PA elimination occurs only after treatment, that assumption allows direct comparison of the composite modified Gompertz/second exponential growth model parameters between untreated and treated rabbits.

It should be noted that measured plasma PA (free plus raxibacumab bound) levels are decreased in the presence of raxibacumab, with the expected peak raxibacumab level resulting in measured plasma PA concentrations about 30% to 64% of the actual value. Hence, treatment (levofloxacin alone *versus* levofloxacin plus raxibacumab) was assessed as a possible covariate in the model. Body weight, sex, age, size of spore challenge, duration of spore challenge, and TBAC were also evaluated as potential covariates.

## 3. Results and Discussion

### 3.1. Spore Challenge, Treatment, and Survival Outcome

A total of 180 rabbits were challenged with a target 200 × LD_50_ *B. anthracis* (Ames strain) spore dose (range: 86 to 348 × LD_50_). There were no significant differences in spore exposure between treatment groups or among challenge days. Of the 180 rabbits that were challenged, 104 rabbits (58%, 54 males and 50 females) died prior to the 84 h post-challenge treatment time (the untreated group), while 76 rabbits (42%) remained alive and were randomized to treatment with levofloxacin and placebo (*n* = 37, 18 males and 19 females) or levofloxacin and raxibacumab (*n* = 39, 18 males and 21 females). At 28 days after the last levofloxacin dose, there were 24 survivors (65%) in the levofloxacin/placebo group and 32 survivors (82%) in the raxibacumab/levofloxacin group. Although the 17% absolute difference in survival rate between the groups did not reach statistical significance (*p* = 0.0874), that difference in mortality rate is clinically meaningful, since there were about half as many deaths (18% *versus* 35%) when raxibacumab was administered. There was no evidence of any relationship between survival time or outcome and body weight, sex, age, size of spore challenge, duration of spore challenge, or time to first bacteremia by culture.

It should be noted that the survival rate for rabbits that were treated with levofloxacin alone (65%) approximates the 55% survival rate for humans in the 2001 anthrax attack [3,24]. Indeed, the reason for selection of the 84 h treatment time was to attain a similar survival rate as for the 2001 attack with antibiotic alone, while using a levofloxacin dose that resulted in exposures in rabbits that are similar to those for humans administered levofloxacin. The 50 mg/kg levofloxacin dose in rabbits did attain similar peak and trough drug levels as for humans, per the levofloxacin product label (data not shown). Since antibiotic exposure could not be reduced, the delayed treatment time was necessary to match the survival rate in the 2001 anthrax attack.

### 3.2. PA Kinetics in Untreated Rabbits

**Figure 1:**
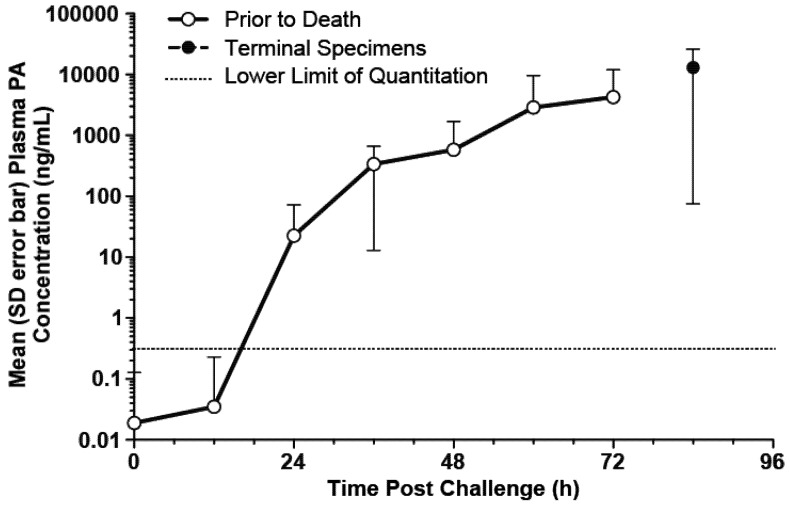
Mean (SD error bars) plasma PA concentration-time profiles in untreated rabbits after a targeted 200 × LD_50_ (Ames strain) inhaled *B. anthracis* spore challenge. For the purposes of this figure, the terminal specimen results are plotted at the 84h post-challenge time, without regard to the actual collection times for those specimens. All untreated rabbits died prior to the 84h-post challenge time.

The mean (with SD error bars) observed plasma PA concentration-time profiles for the untreated animals included in the analyses are illustrated in Figure 1. The profiles for individual animals generally followed the pattern of initial lag phase, a rising phase, a plateau, and then another rising phase, a pattern that has been reported previously in this animal model [17]. The exceptions were that some animals died prior to attaining either the plateau phase or the second rising phase. A composite modified Gompertz/second exponential growth model, consisting of the Gompertz model with a second exponential growth phase following a second lag phase, was consistent with the observed data.

As shown in Figure 1, there was variability in the concentrations for the terminal specimens. Due to the large variability of the terminal specimen results, and uncertainty concerning the contribution of post-mortem changes to that variability, the terminal specimen results were excluded from the PA kinetic modeling.

Modeling of inter-individual variability for all of the model parameters was attempted, but was not successful. In the final model, inter-individual variability could not be estimated for PA concentration at time 0 (*N_0_*), lag time for the first growth phase (λ), maximum specific growth rate for the first phase (μ_m_), or lag time for the second growth phase (λ_2_). Given the complexity of this model, the substantial variability in the data, and the varying contribution of each animal to the model, inability to adequately model inter-individual variability for all parameters in the model could be expected. Rather than revealing any deficiency in the model, this outcome may be a qualitative reflection of the data being modeled. Since the purpose of this modeling exercise was to provide a description of the observed data, the modeling results can be considered adequate for the intended purpose, but should be interpreted with caution due to the poor definition of the variability components.

**Figure 2:**
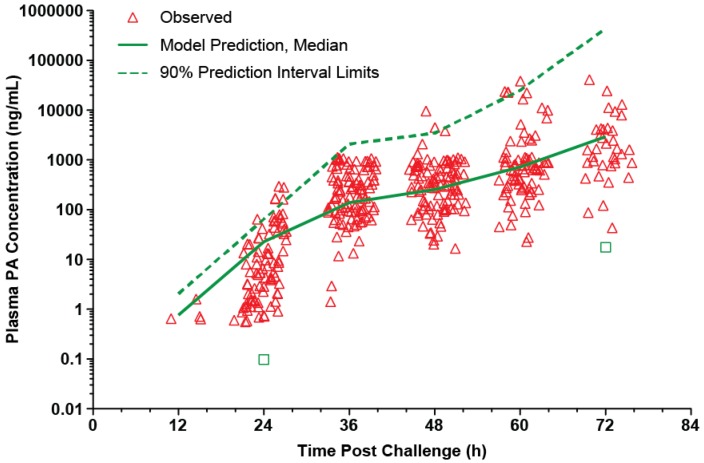
Visual predictive check for PA kinetic model with individual observed plasma PA concentrations in untreated rabbits after a targeted 200 × LD_50_ (Ames strain) inhaled *B. anthracis* spore challenge. The lower bound of the 90% prediction interval is zero at all post-challenge times, except 24 and 72h, which are marked as green squares. A composite modified Gompertz/second exponential growth phase following a second lag phase model was used for nonlinear mixed effects modeling (see Materials and Methods section), while the median prediction and prediction interval were generated by running the final model in simulation mode, generating 200 replicate simulations for each rabbit.

The model was evaluated using a visual predictive check. As shown in Figure 2, the majority of the observed plasma PA concentrations are within the 90% prediction interval, suggesting that the model describes the data well. The parameter estimates for the model are summarized in Table 2.

**Table 2:** PA kinetic parameters in untreated rabbits after a targeted 200 × LD_50_ (Ames strain) inhaled *B. anthracis* spore challenge, based on fitting of a composite modified Gompertz/second exponential growth phase following a second lag phase model (see Materials and Methods section) to the data.

Parameters	Mean ^1^	CV% ^1^
*N_0_* (ng/mL)	0.38 (20.5%)	- ^2^
Association of TBAC with *N_0_* ^3^	*N_0_* × (TBAC/34)^−3.55^ (28.2%)	
At 10.57 h	24.08	
At 20 h	2.51	
At 30 h	0.59	
At 34 h	0.38	
At 40 h	0.21	
At 44.72 h	0.14	
λ (h)	20 (2.7%)	- ^2^
μ_m_ (h^−1^)	0.86 (9.8%)	- ^2^
A (unitless)	5.08 (5.1%)	24.5 (29.9%)
λ_2_ (h)	22 (0.1%)	- ^2^
μ_m,2_ (h^−1^)	0.16 (3.4%)	30.4 (21.1%)
Residual variability (CV%)	61.2 (7.4%)	

Abbreviations: CV%, coefficient of variation; *N_0_*, PA concentration at time 0; A, natural log of the ratio of the PA concentration in the asymptotic phase to *N_0_*; μ_m_, maximum specific growth rate for the first phase; λ, lag time for the first phase; λ_2_, lag time for the second growth phase; μ_m,2_, maximum specific growth rate for the second phase; TBAC, time to first positive bacteremia by culture.

^1^ Values in parentheses represent the relative standard error of the estimate.

^2^ Model did not include inter-individual variability for this parameter.

^3^ TBAC was normalized to a value of 34 h.

As shown in Table 2, lower *N_0_* values were associated with longer TBAC values. That association is intuitive since higher values of *N_0_* would be expected to reflect earlier and more abundant spore germination and vegetative growth, leading to earlier detection of bacteremia. No other associations between PA kinetics and the covariates assessed were found.

**Figure 3:**
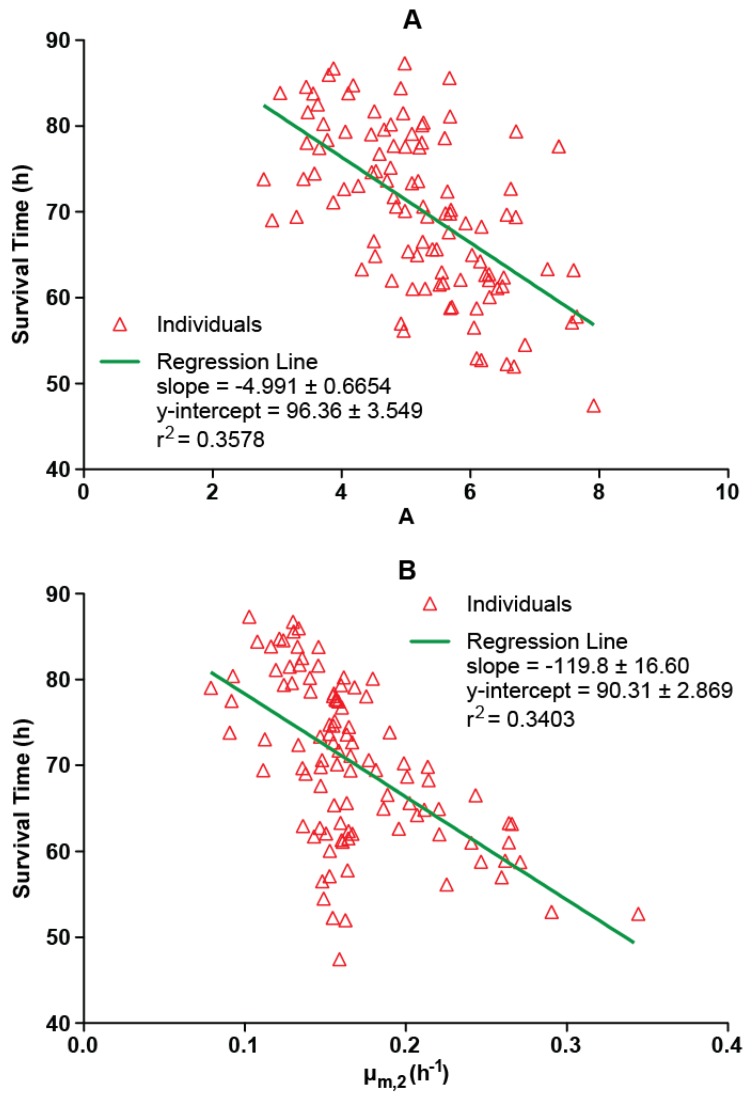
Relationships between survival time and natural log of the ratio of the PA concentration in the asymptotic (plateau) phase to *N_0_* (**A**) and maximum specific growth rate for the second phase (**B**) in untreated rabbits after a targeted 200 × LD_50_ (Ames strain) inhaled *B. anthracis* spore challenge. Data points represent the *post hoc* PA kinetic parameter estimates for individual rabbits obtained from fitting the observed data to a composite modified Gompertz/second exponential growth phase following a second lag phase model (see Materials and Methods section).

Possible relationships between survival time and the natural log of the ratio of the PA concentration in the asymptotic (plateau) phase to *N_0_* (A) or the maximum specific growth rate for the second phase (μ_m,2_) were assessed (Figure 3). Survival times tended to become shorter as A increased, with A accounting for 36% of the variability in the survival time data. This is intuitive since higher plateau PA concentrations would be expected to be associated with increased morbidity. Survival time also tended to decrease as μ_m,2_ increased, with μ_m,2_ explaining 34% of the variability in survival times. Since higher values of μ_m,2_ represent more rapid rates of increase in PA concentrations during the second phase of the profile, this relationship could be expected. Since the relationships between survival time and magnitude of PA levels or rate of increase of PA levels are not entirely predictive, it is likely that survival time was partially dependent on other factors. 

### 3.3. PA Kinetics in Treated Rabbits

**Figure 4:**
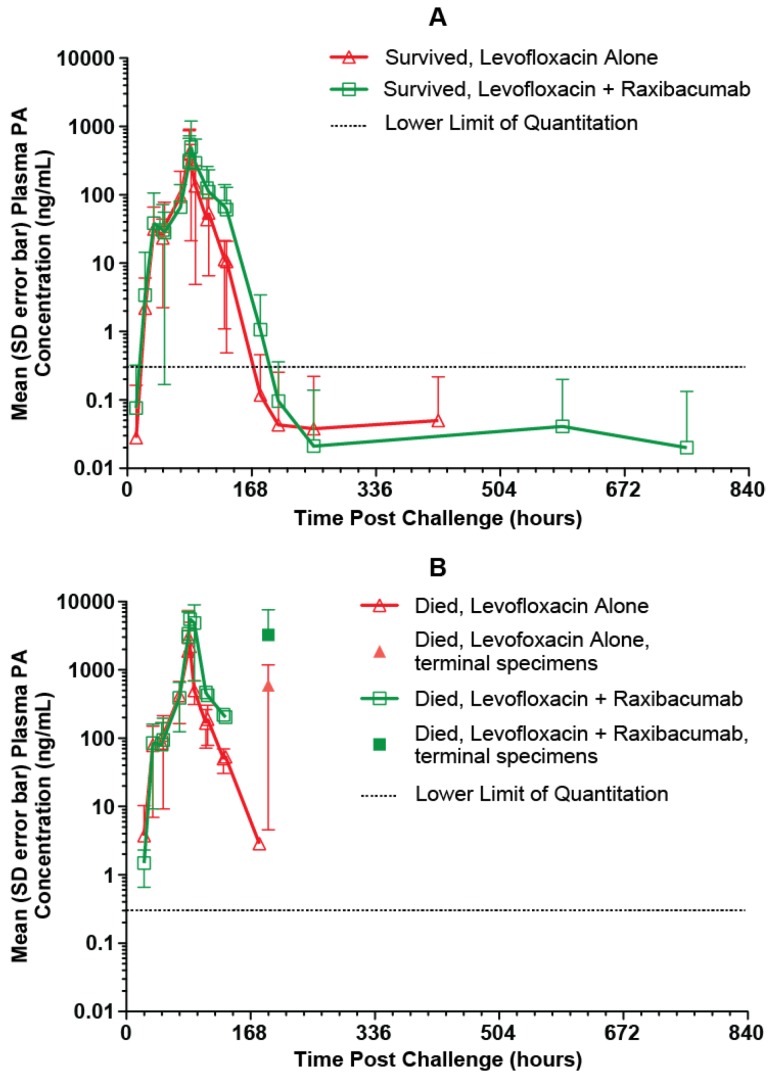
Mean (SD error bars) plasma PA concentration-time profiles in rabbits that survived (**A**) and rabbits that died (**B**) after a targeted 200 × LD_50_ (Ames strain) inhaled *B. anthracis* spore challenge, followed by treatment with IG 50mg/kg qd×3 levofloxacin doses with a single intravenous placebo or 40mg/kg raxibacumab dose after the first levofloxacin dose. For the purposes of this figure, the terminal specimen results are plotted at the 192h post challenge time, without regard to the actual collection times for those specimens.

The mean (with SD error bars) observed plasma PA concentration-time profiles for the treated animals are illustrated in Figure 4. For rabbits that survived, the mean plasma PA concentration-time profile had an initial rising phase, and after the treatment time, when bacteremia had been eradicated (data not shown), plasma PA concentrations declined. This type of PA profile was characteristic of all surviving animals. The plasma PA concentration-time profiles for treated rabbits that died were more varied. Some treated rabbits that died had plasma PA concentration-time profiles that generally exhibited increasing concentrations (4 of 20 rabbits [20%]), while others had profiles in which plasma PA concentrations were decreasing at the time of death (16 of 20 rabbits [80%]). It is possible that the rabbits whose profiles exhibited only increasing concentrations died before clearance of PA became evident. For the rabbits that died during the period when plasma PA levels were decreasing, it is likely that death occurred as a result of cumulative organ damage from the toxin or host specific response to the bacterium, despite the evidence that clearance of PA was ongoing at the time of death.

The data for treated animals were consistent with the composite modified Gompertz/second exponential growth model with a monoexponential elimination phase. Alternate parameterizations using clearance and volume of distribution terms and/or a delay time between treatment and the start of PA elimination were unsuccessful due to apparent over-parameterization of the model. In particular, the estimate for the delay time was very small, implying that the data did not support estimation of that parameter. The final model was parameterized with a first-order elimination rate constant (k_elm_) and no delay time.

Modeling of inter-individual variability for all of the model parameters was attempted, but was not successful. In the final model, inter-individual variability could not be estimated for λ, μ_m_, *N_0_*, μ_m,2_, and k_elm_. Given the complexity of this model and the substantial variability in the data, inability to adequately model inter-individual variability for all parameters in the model could be expected. Rather than revealing any deficiency in the model, this outcome may be a qualitative reflection of the data being modeled. Since the purpose of this modeling exercise was to provide a description of the observed data, the modeling results can be considered adequate for the intended purpose, but should be interpreted with caution due to the poor definition of the variability components.

**Figure 5:**
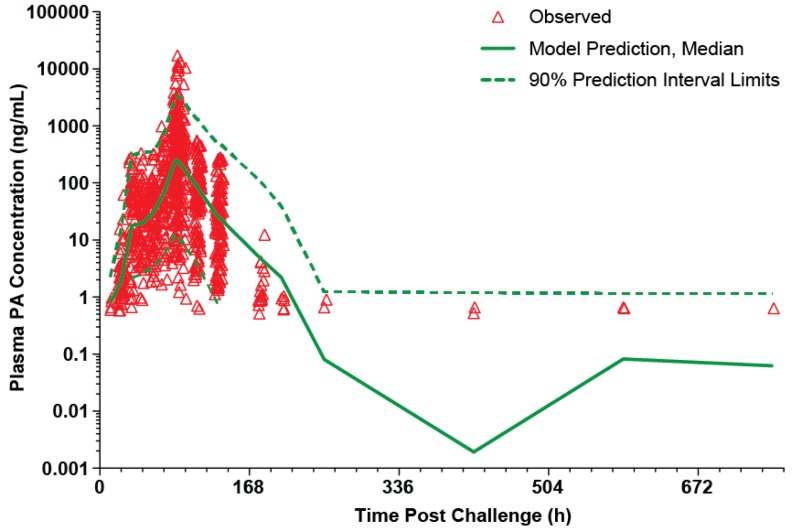
Visual predictive check for PA kinetic model with individual observed plasma PA concentrations in treated rabbits after a targeted 200 × LD_50_ (Ames strain) inhaled *B. anthracis* spore challenge, followed by treatment with IG 50mg/kg qd×3 levofloxacin doses with a single intravenous placebo or 40mg/kg raxibacumab dose after the first levofloxacin dose. The lower bound of the 90% prediction interval is zero at post-challenge times greater than 168h. A composite modified Gompertz/second exponential growth phase following a second lag phase/monoexponential elimination phase was used for nonlinear mixed effects modeling (see Materials and Methods section), while the median prediction and prediction interval were generated by running the final model in simulation mode, generating 200 replicate simulations for each rabbit.

The model for the treated rabbits was evaluated using a visual predictive check. As shown in Figure 5, the majority of the observed plasma PA concentrations are within the 90% prediction interval, suggesting that the model describes the data well. The appearance of the predicted profile at times greater than 336 h post-challenge is driven by the relatively few rabbits that had measurable PA concentrations at those times. The parameter estimates for the model are summarized in Table 3.

Possible differences in PA kinetics for treated rabbits that died and survived were assessed (Table 4). The 95% confidence interval (CI) of the *post hoc* PA kinetic parameter estimates for the treated rabbits that survived and those that died generally overlapped, indicating no statistically significant differences, with the exceptions of A and λ_2_. For the treated rabbits that died, plateau PA levels (A) were significantly higher (33%) than those for rabbits that survived, and duration of the plateau phase was significantly shorter (29%) than for rabbits that survived. These findings are consistent with the hypothesis that death due to toxemia tends to be associated with PA levels that are higher, and progress to higher levels earlier.

As shown in Table 3, some variability in λ was associated with both sex (SEX) and treatment group (GRP), while some variability in μ_m_ was associated with sex, and some variability in k_elm_ was associated with treatment group. No other associations between PA kinetics and the covariates assessed were found. For females, λ was about 80% of that for males, and μ_m_ was only about 40% of that in males. Despite these differences, there was not a difference in survival rates between males and females, suggesting that the differences were not clinically meaningful. For rabbits, administered levofloxacin plus raxibacumab, λ was 7% longer than that for rabbits administered levofloxacin alone. This small difference would not be expected to be clinically meaningful. PA elimination was 45% slower in rabbits administered levofloxacin with raxibacumab than in rabbits administered levofloxacin alone. This may reflect systemic persistence of measurable PA resulting from the PA-raxibacumab complex. However, λ and k_elm_ were not significantly different between the survivor and non-survivor subgroups (Table 4), implying that the effects of the treatment group on those parameters were not clinically meaningful.

**Table 3:** PA kinetic parameters in treated rabbits after a targeted 200 × LD_50_ (Ames strain) inhaled *B. anthracis* spore challenge, followed by treatment with IG 50mg/kg qd×3 levofloxacin doses with a single intravenous placebo or 40mg/kg raxibacumab dose after the first levofloxacin dose, based on the fitting of a composite modified Gompertz/second exponential growth phase following a second lag phase/monoexponential elimination phase model (see Materials and Methods section) to the data.

Parameters			Mean ^1^	CV% ^1^
*N_0_* (ng/mL)			0.83 (11.9%)	- ^2^
λ (h)			24 (1.2%)	- ^2^
	Effect of treatment group on λ (h)^3^		λ × 1.07^GRP^ (1.1%)	
		Levofloxacin alone	24	
		Levofloxacin plus raxibacumab	26	
	Effect of sex on λ (h) ^4^		λ × 0.797^SEX^ (4.2%)	
		Male	24	
		Female	19	
μ_m_ (h^−1^)			1.55 (16.7%)	- ^2^
	Effect of sex on μ_m_ (h^−1^) ^4^		μ_m_× 0.409^SEX^ (29.6%)	
		Male	1.55	
		Female	0.63	
A (unitless)			3.16 (6.4%)	39.0 (16.8%)
λ_2_ (h)			50 (4.9%)	24.1 (20.6%)
μ_m,2_ (h^−1^)			0.16 (5.8%)	- ^2^
k_elm_ (h^−1^)			0.067 (3.5%)	- ^2^
	Effect of treatment group on k_elm_ (h^−1^) ^3^		k_elm_× 0.547^GRP^ (7.1%)	
		Levofloxacin alone	0.067	
		Levofloxacin plus raxibacumab	0.037	
Residual variability (CV%)			46.2% (7.5%) CV% for proportional error component	
			76.7 ng/mL (34.2%) SD for additive error component	

Abbreviations: CV%, coefficient of variation; *N_0_*, plasma PA concentration at time 0; A, natural log of the ratio of the PA concentration in the asymptotic phase to *N_0_*; μ_m_, maximum specific growth rate for the first phase; λ, lag time for the first phase; λ_2_, lag time for the second growth phase; μ_m,2_, maximum specific growth rate for the second phase; k_elm_, PA elimination rate constant; GRP, treatment group.

^1^ Values in parentheses represent the relative standard error of the estimate.

^2^ Model did not include inter-individual variability for this parameter.

^3^ For treatment coded as 0 for levofloxacin alone and as 1 for levofloxacin plus raxibacumab.

^4^ For sex coded as 0 for males and as 1 for females.

**Table 4:** Summary of *post hoc* PA kinetic parameter estimates for treated rabbits that survived and died after a targeted 200 × LD_50_ (Ames strain) inhaled *B. anthracis* spore challenge, followed by treatment with IG 50mg/kg qd×3 levofloxacin doses with a single intravenous placebo or 40mg/kg raxibacumab dose after the first levofloxacin dose. Mean and 95% CI of the *post hoc* PA kinetic parameter estimates for individual rabbits in each subgroup are displayed, based on fitting the observed data to a composite modified Gompertz/second exponential growth phase following a second lag phase/monoexponential elimination phase model (see Materials and Methods section).

	λ	μ_m_	A	*N_0_*	λ_2_	μ_m,2_	k_elm_
	(h)	(h^−1^)	Unitless	(ng/mL)	(h)	(h^−1^)	(h^−1^)
Rabbits That Died							
*N*	20	20	20	20	20	20	20
Mean	22	1.09	4.14	0.83	39	0.16	0.057
95% CI	(21, 23)	(0.87, 1.31)	(3.60, 4.68)	(0.83, 0.83)	(36, 42)	(0.16,0.16)	(0.050, 0.063)
Rabbits That Survived							
*N*	56	56	56	56	56	56	56
Mean	22	1.06	3.12	0.83	55	0.16	0.050
95% CI	(22, 23)	(0.94, 1.18)	(2.83, 3.40)	(0.83, 0.83)	(52, 58)	(0.16, 0.16)	(0.046, 0.054)

Abbreviations: *N_0_*, PA concentration at time 0; A, natural log of the ratio of the PA concentration in the asymptotic phase to *N_0_*; μ_m_, maximum specific growth rate for the first phase; λ, lag time for the first growth phase; λ_2_, lag time for the second growth phase; μ_m__,2_, maximum specific growth rate for the second phase; k_elm_, PA elimination rate constant; CI, confidence interval.

Based on the k_elm_ of PA, the half-lives of PA elimination (*t_1/2.elm_*) in this study were 10 and 19 h for rabbits administered levofloxacin alone and levofloxacin with raxibacumab, respectively, with the difference likely reflecting persistence of the PA-raxibacumab complex in circulation. That is, when raxibacumab is not present to bind PA, the PA *t_1/2.elm_* is 10 h. However, when raxibacumab is present (and raxibacumab has a half-life of ~19 days), PA is bound in PA-raxibacumab complexes. Although the t_1/2.elm_ of the complex has not been measured, it is very likely extremely long (many days) relative to that for PA alone (less than 0.5 day). In essence, the PA-raxibacumab complex probably acts as a sink which slows PA elimination. The formation of the complex also clears plasma of the toxin, leading to efficacy.

There was similarity in PA kinetics between the untreated rabbits (*i.e.*, those that died prior to the 84 h post-spore challenge treatment time; Table 2) and the treated rabbits (Table 3), with the exception of λ_2_, which essentially represents the duration of the plateau phase of the serum PA profile. This suggests that although an inhaled *B. anthracis* spore challenge can be lethal, survival to the 84 h post-challenge treatment time was dependent on the duration of the plateau phase, rather than other PA kinetics. Since λ_2_ was not associated with the covariates assessed in this study, survival to the 84 h post-challenge treatment time was likely dependent on other factors. Most important, for those animals that were treated in the late stages of toxemia, both levofloxacin alone and levofloxacin plus raxibacumab were highly effective in attaining high survival rates.

Overall, this study provides a detailed examination of PA kinetics in a large number of untreated rabbits, as well as in rabbits treated with antibiotic alone or antibiotic plus the antitoxin raxibacumab, when treatment was initiated late in the course of anthrax disease. Despite the limitations of predicting survival time, the assessment of PA kinetics in untreated rabbits does reveal that death is associated with the highest plasma PA concentration attained for an animal, and that survival time is a function of how rapidly PA levels increase and how high the levels become, although survival time is influenced by other factors not identified in this study. Relative to this, the PA kinetics observed in this study were consistent with those observed in a smaller prior study of untreated rabbits [17]. The PA kinetics model for treated rabbits extends the model for untreated rabbits to account for the disappearance of systemic PA that occurs once bacteremia has been eradicated. These models may be useful for design of studies to further probe the factors that influence survival time or responses to alternate treatment regimens.

## 4. Conclusions

The 17% absolute difference in survival rate between the treated groups did not reach statistical significance (*p* = 0.0874). However, that difference was clinically meaningful, with about half as many deaths (18% *versus* 35%) when raxibacumab was administered.

Plasma PA concentration-time profiles in untreated rabbits that died could be fit to a diauxic model (rise-plateau-rise). For that group, TBAC was associated with *N_0_*, the initial PA exposure, such that longer TBAC associated with lower values for *N_0_*. Survival time was partially dependent on plateau PA concentrations, with higher plateau PA concentrations relating to shorter survival times, and was also partially dependent on rate of increase in PA concentrations in the second phase, with higher rates of increase relating to shorter survival times.

Rabbits that survived had plasma PA concentrations that tended to decrease at times coincident with negative bacteremia. In treated rabbits, treatment group was a significant covariate for λ and k_elm_, while sex was a significant covariate for λ and μ_m_. None of these effects were clinically meaningful, since they were not associated with differences in survival outcome.

In surviving rabbits, the *t*_1/2_ for PA was 10 and 19 h for rabbits administered levofloxacin alone and levofloxacin plus raxibacumab, with the difference likely reflecting persistence of the PA-raxibacumab complex in the circulation. For the treated rabbits that died, plateau PA levels were higher than in surviving rabbits and duration of the plateau phase was shorter than in surviving rabbits, consistent with death due to toxemia being associated with PA levels that are higher, and progress to higher levels earlier.

PA kinetics were similar between untreated rabbits (*i.e.*, those that died prior to the 84 h post-spore challenge treatment time) and the treated rabbits that survived, with one exception: the duration of the plateau phase, reflected by λ_2_, was nearly twice as long in treated than untreated rabbits.
